# Modulation of striatal functional connectivity differences in adults with and without autism spectrum disorder in a single-dose randomized trial of cannabidivarin

**DOI:** 10.1186/s13229-021-00454-6

**Published:** 2021-07-01

**Authors:** Charlotte M. Pretzsch, Dorothea L. Floris, Bogdan Voinescu, Malka Elsahib, Maria A. Mendez, Robert Wichers, Laura Ajram, Glynis Ivin, Martin Heasman, Elise Pretzsch, Steven Williams, Declan G. M. Murphy, Eileen Daly, Gráinne M. McAlonan

**Affiliations:** 1 Department of Forensic and Neurodevelopmental Sciences, Institute of Psychiatry, Psychology and Neuroscience, King’s College London, 16 De Crespigny Park, London, SE5 8AF UK; 2 Department of Cognitive Neuroscience, Donders Institute for Brain, Cognition and Behaviour, Radboud University Nijmegen Medical Centre, Nijmegen, The Netherlands; 3 Department of Liaison Psychiatry, Bristol Royal Infirmary, University Hospitals Bristol and Weston NHS Foundation Trust, Bristol, UK; 4 Instituto de Investigación Sanitaria Gregorio Marañón, Madrid, Spain; 5 Department of Psychiatry GGZ Geest, Amsterdam, The Netherlands; 6 Medicines Discovery Catapult, Alderley Park, Alderley Edge, SK10 4TG Cheshire UK; 7 South London and Maudsley NHS Foundation Trust Pharmacy, London, UK; 8 Department of General, Visceral, and Transplant Surgery, Ludwig-Maximilians-University Munich, Munich, Germany; 9 Department of Neuroimaging Sciences, Institute of Psychiatry, Psychology and Neuroscience, King’s College London, London, UK

**Keywords:** Autism spectrum disorder, Autism spectrum condition, Cannabidivarin, CBDV, Functional connectivity, Striatum

## Abstract

**Background:**

Autism spectrum disorder (ASD) has a high cost to affected individuals and society, but treatments for core symptoms are lacking. To expand intervention options, it is crucial to gain a better understanding of potential treatment targets, and their engagement, in the brain. For instance, the striatum (caudate, putamen, and nucleus accumbens) plays a central role during development and its (atypical) functional connectivity (FC) may contribute to multiple ASD symptoms. We have previously shown, in the adult autistic and neurotypical brain, the non-intoxicating cannabinoid cannabidivarin (CBDV) alters the balance of striatal ‘excitatory–inhibitory’ metabolites, which help regulate FC, but the effects of CBDV on (atypical) striatal FC are unknown.

**Methods:**

To examine this in a small pilot study, we acquired resting state functional magnetic resonance imaging data from 28 men (15 neurotypicals, 13 ASD) on two occasions in a repeated-measures, double-blind, placebo-controlled study. We then used a seed-based approach to (1) compare striatal FC between groups and (2) examine the effect of pharmacological probing (600 mg CBDV/matched placebo) on atypical striatal FC in ASD. Visits were separated by at least 13 days to allow for drug washout.

**Results:**

Compared to the neurotypicals, ASD individuals had lower FC between the ventral striatum and frontal and pericentral regions (which have been associated with emotion, motor, and vision processing). Further, they had higher intra-striatal FC and higher putamenal FC with temporal regions involved in speech and language. In ASD, CBDV reduced hyperconnectivity to the neurotypical level.

**Limitations:**

Our findings should be considered in light of several methodological aspects, in particular our participant group (restricted to male adults), which limits the generalizability of our findings to the wider and heterogeneous ASD population.

**Conclusion:**

In conclusion, here we show atypical striatal FC with regions commonly associated with ASD symptoms. We further provide preliminary proof of concept that, in the adult autistic brain, acute CBDV administration can modulate atypical striatal circuitry towards neurotypical function. Future studies are required to determine whether modulation of striatal FC is associated with a change in ASD symptoms.

**Trial registration:**

clinicaltrials.gov, Identifier: NCT03537950. Registered May 25th, 2018—Retrospectively registered, https://clinicaltrials.gov/ct2/show/NCT03537950?term=NCT03537950&draw=2&rank=1.

**Supplementary Information:**

The online version contains supplementary material available at 10.1186/s13229-021-00454-6.

## Background

Autism spectrum disorder (ASD) is a neurodevelopmental condition. Core symptoms of ASD include difficulties across the domains of social communication and restricted and repetitive behaviours, and altered sensory processing [1]. Additional transdiagnostic symptoms comprise, e.g. atypical motor [2], (social) reward [3], executive [4], mood, and emotion [5, 6] processing. Combined, these difficulties incur a high cost to affected individuals and society [7–9]. Nonetheless, there are currently no effective pharmacological treatments for the core symptoms of ASD, and conventional drug treatment of co-occurring difficulties is often unsatisfactory. To expand intervention options in ASD, it is crucial to gain a better understanding of potential brain targets and their engagement by putative treatments.

One potential target is the striatum, a basal ganglia structure comprising the caudate, putamen, and nucleus accumbens [10]. The striatum is functionally connected to widespread cortical domains and contributes to nearly every cognitive-behavioural function altered in ASD, such as social [11], motor [12], or reward [11] processing. For instance, the caudate and accumbens connect with the cortex along a ventral-dorsal limbic-cognitive control axis [13], whereas putamenal functional connectivity (FC) with cortex follows a rostral-caudal executive control-motor axis [13–15]. Also, the striatum serves a critical role throughout brain development. For example, a recent study in > 900 children and adults reported an association between (dys)maturation of striatal FC and pathophysiology in the general population [16]. This suggests that striatal impairment may disturb neurodevelopment in general and contribute to conditions such as ASD.

Consistent with this, previous studies have reported multiple atypicalities of the striatum and its FC in ASD. These include, for example, an imbalance in the levels of excitatory glutamatergic and inhibitory GABAergic metabolites in children and adults [17–19]; hypoactivation during response-shifting [20] and social (reward) processing [21] (and the latter correlated with ASD symptom severity in adolescents [21]); and volumetric expansion of the caudate [22–24], which correlated with repetitive behaviour in adults [23]. Our previous work also found that volumetric expansions are present from at least as early as 6 months in infants at risk of developing ASD, and a larger striatum at this age predicted autistic symptoms and diagnosis at 36 months [25]. This highlights the relevance of the striatum in the pathophysiology of ASD and suggests that striatal pathology may be primary to ASD, rather than acquired as a secondary consequence of living with this condition.

Therefore, it is perhaps unsurprising that a wealth of research has found striatal FC to be altered in ASD, but the direction of findings varies considerably, with some striatal regions being hypoconnected and others being hyperconnected with the rest of the brain [26–30]. While the heterogeneous nature of participating cohorts likely contributes to frequent replication difficulties, previous studies have also relied primarily upon relatively ‘coarse’ definitions of the complex striatal circuitry. Thus, more fine-grained analyses are needed to dissect striatal FC differences in ASD and to test whether these constitute a biological target that can be ‘shifted’ pharmacologically in ASD.

Therefore, in the current study, we applied a detailed seed-based approach [13, 31, 32] to resting state functional magnetic resonance imaging data with the aim of examining the FC of the striatum in autistic and neurotypical adults. Based on previous findings [26–30], we predicted a complex pattern of both striatal hyper- and hypoconnectivity with the rest of the brain in ASD relative to a neurotypical control group. Our second aim was to test whether (disrupted) striatal FC could be engaged by a candidate treatment. Promising candidates include non-intoxicating cannabinoids such as cannabidivarin (CBDV). Emerging evidence suggests that CBDV acts on multiple neuroglial targets, which may effect a downstream modulation of the brain excitation–inhibition (E–I) balance, a crucial regulator of FC [33–35]. For instance, preclinical studies have shown that CBDV binds to several transient receptor potential (TRP) receptors (e.g. vanilloid type 1 (TRPV1), vanilloid type 2 (TRPV2), and ankyrin type 1 (TRPA1) [36]). These receptors have been identified on (sub)cortical excitatory and inhibitory neurons [37] and microglia [38], including in the striatum [39, 40]. Given the role of the striatum as a key neural hub, CBDV could alter excitatory and inhibitory neurotransmission—and hence striatal FC—directly through these striatal receptors and/or indirectly through action on interconnected regions. In line with this, we have previously demonstrated that a single dose of CBDV alters striatal levels of glutamate [18]. However, whether CBDV also shifts striatal FC remains to be investigated. Hence, here we examined and compared striatal FC in the adult autistic and neurotypical brain at baseline (placebo) and following the administration of a single dose of CBDV (600 mg).

## Methods

### Procedure

This research was conducted in accordance with the Declaration of Helsinki, at the Institute of Psychiatry, Psychology and Neuroscience at De Crespigny Park, SE5 8AF, London, UK (August 2016 to February 2017). The King’s College London Research Ethics Committee provided institutional ethical approval for this study (reference HR15/162744). The Medicines and Health Research Authority in the UK confirmed that our study design was not a clinical trial. Nonetheless, in the interests of transparency, we registered this experimental study on clinicaltrials.gov (identifier: NCT03537950, entry name: HR15-162744; Registered May 25th, 2018—retrospectively registered, https://clinicaltrials.gov/ct2/show/NCT03537950?term=NCT03537950&draw=2&rank=1). All participants provided written informed consent and took part in all aspects of this case–control observational study.

This project was a placebo-controlled, randomized, double-blind, repeated-measures, cross-over case–control study as part of a larger investigation into the role of phytocannabinoids in ASD; clinicaltrials.gov (identifier: NCT03537950, entry name: HR15-162744). Accordingly, the sample size was determined using power analyses to achieve sufficient power for this larger investigation [17]. Drugs were allocated in a pseudo-randomized order, so that each participant received each compound (placebo, PLC; cannabidivarin, CBDV) once. Approximately half of our participants received PLC first, and half CBDV. This randomization was implemented by G. M. M. using https://www.random.org/. All participants attended for two visits, which were separated by at least 13 days to allow for drug wash-out (between-visit times were consistent across visits and participants). Data acquisition from both groups occurred during the same time period. On each visit, participants received a brief health check, a liquid oral dose of the pharmacological probe (600 mg of CBDV; in line with previous single-dose studies of CBD in adults (e.g. [41]) or a matched placebo [baseline], both provided by GW Research Ltd, Cambridge, UK), and a second brief health check to test for potential acute adverse reactions/side effects. Two hours after drug administration (at the time of peak plasma levels [Investigator’s brochure for CBDV, Edition 4, May 2016]), participants underwent scanning, followed by a third health check to ensure that they experienced no ill effects and were fit to leave the department.

### Participants

Potential participants were excluded if they had a comorbid major psychiatric or medical disorder affecting brain development (e.g. schizophrenia or epilepsy), a history of head/brain injury, a genetic disorder associated with ASD (such as fragile X syndrome or tuberous sclerosis), or an IQ below 70. We also excluded participants if they were reliant on regular medication known to affect directly the glutamate and GABA neurotransmitter systems, such as benzodiazepines, but included those on other medications frequently prescribed in ASD. Participants were asked to abstain from using cannabis and/or other illicit substances in the month prior and during the study, and from drinking alcohol on the days before visits. All participants in the ASD group had a clinical diagnosis of ASD made according to ICD10 research criteria, supported by the use of standardized research diagnostic instruments (Autism Diagnostic Observation Schedule, ADOS; and Autism Diagnostic Interview-Revised, ADI-R) [42–44]. For more information, please refer to Table 1.

**Table 1:** Participant demographics

Measure	ASD (*n* = 13)	Neurotypicals (*n* = 15)	Test statistic
Age at visit 1 (years)	30.6 ± 11.2 [20–50]	28.5 ± 6.1 [21–40]	*F*(1, 26) = 0.389, *p* = .538
ADI com^a^	7.5 ± 5.0		
ADI rep^a^	4.0 ± 2.6		
ADOS com	4.4 ± 3.3		
ADOS soc	7.6 ± 4.1		
AQ^a^	29.1 ± 13.7		
FSIQ	113.0 ± 18.1	125.3 ± 12.7	*F*(1, 26) = 4.421, *p* = .045
*T*_V1–V2_ (days)	31.2 ± 17.9	28.1 ± 15.0	*F*(1, 26) = 0.248, *p* = .623
Mean motion (CBDV)	0.015 ± 0.067	0.008 ± 0.043	*F*(1, 26) = 0.100, *p* = .754
Mean motion (PLC)	0.014 ± 0.049	0.008 ± 0.050	*F*(1, 26) = 0.097, *p* = .758

Demographics (mean ± standard deviation [range])) and test statistic of between-group comparison

*ADI* autism diagnostic interview (com, communication domain; rep, restricted and repetitive behaviours domain), *ADOS* autism diagnostic observation schedule (com, communication domain; soc, social domain), *AQ* autism quotient, *CBDV* cannabidivarin, *FSIQ* full-scale intelligence quotient, *PLC* placebo, *T*_*V1*–*V2*_, time between visits 1 and 2

^a^*n* = 10

### Image data acquisition

All imaging data were acquired on a 3 T GE Excite II magnetic resonance imaging (MRI) scanner (GE Medical Systems, Milwaukee, WI, USA). The scanning protocol included a structural MRI scan acquired using a 3D inversion recovery prepared fast spoiled gradient recalled (IR-FSPGR) sequence (slice thickness = 1.1 mm, spatial positions = 124, flip angle = 20°, field of view (FoV) = 280 mm, echo time (TE) = 2.844 ms, repetition time (TR) = 7.068 ms, inversion time (TI) = 450 ms, matrix = 256 × 256). This structural MRI scan was used for co-registration of the functional volumes. The scanning protocol also included a resting state MRI scan. This scan was acquired using an echo-planar imaging (EPI) sequence (slice thickness = 3 mm, slice gap = 3.3 mm, flip angle = 75°, FoV = 240 mm, TE = 30 ms, TR = 2000 ms, TI = 0 ms). We collected data for 256 time points, i.e. the resting state scan lasted 512 s.

### Data processing

All analyses were performed using in-house software, CONN v.18b [45] and MATLAB R2018b (The MathWorks, Inc., MA, USA).

### Structural data processing

All T1-weighted structural MRI scans were inspected manually to ensure adequate data quality and signal-to-noise ratio and normalized to Montreal Neurological Institute (MNI) space. Next, all volumes were segmented into grey matter (GM), white matter (WM) and cerebrospinal fluid (CSF) to enable the removal of WM and CSF confounds using linear regression.

### Resting state data processing

All T2-weighted resting state MRI scans were inspected manually to exclude data with obvious artefacts, e.g. blurring, distortions, ghosting, or warping.

We discarded the first five functional volumes to allow for magnetization equilibrium. Next, all remaining functional volumes were slice-time corrected, realigned (first: within each subject, all 3D volumes [251 per subject] are aligned to that subject’s first 3D volume (within subject); second: all subjects’ 3D volumes are aligned to the first subject’s first 3D volume (across subjects)) and unwarped. We used ARtifact detection tools (ART) (https://www.nitrc.org/projects/artifact_detect/) to identify functional outliers (global signal z-value threshold = 3 standard deviations and subject motion threshold = 1 mm in line with previous studies, e.g. [46]). Next, we performed functional direct segmentation and normalization to MNI space and smoothed our data using a Gaussian filter with a 6-mm full width at half maximum (FWHM) kernel. Importantly, head motion can distort measures of FC [47]. Therefore, when denoising our data, we not only excluded WM and CSF confounds, but also removed realignment and movement confounds (ART scrubbing and realignment parameters as well as their first-order derivatives) using linear regression. We then filtered (band-pass: 0.008–0.09) and detrended (linear trend removal) our data. We excluded all runs with movement in any dimension ≥ 3 mm/° and/or ≥ 5% of volumes identified as motion outliers (frame-to-frame displacement > 1 mm/° translation/rotation). This resulted in the exclusion of one run (CBDV) from one subject (ASD).

Next, we determined striatal seed regions. As these were based on previous literature and have been validated extensively [13], we followed the previous research approach used in ASD [32] of not customizing them to each individual. Further, given that cortico-striatal FC is known to be hemisphere specific in neurotypicals [48], and given the wealth of research indicating atypical lateralization of FC in ASD [49, 50], we chose to examine seeds separately within each hemisphere rather than merging them. Consequently, our regions of interest included (bilateral) seeds in the caudate: the inferior ventral striatum (Vsi; MNI coordinates: x =  ± 9, y = 9 z = -8), superior ventral striatum (VSs; ± 10 15 0), and dorsal caudate (DC; ± 13 15 9). They also included (bilateral) seeds in the putamen: the dorsal caudal putamen (dcP; ± 28 1 3), dorsal rostral putamen (drP; ± 25 8 6), and ventral rostral putamen (vrP; ± 20 12 -3). In line with previous studies, each of these 12 seeds covered 33.5 voxels (4 mm radius in 2mm^2^ space). For more information on the seed placement, see Additional file 1: Figure S1.

For each subject, we computed whole-brain voxel-wise correlations for the mean time series of each of the 12 seeds. Pearson’s correlation coefficients were Fisher-transformed to improve normality of the data. Individual FC maps were then entered into standard analyses of variance to evaluate experimental effects.

We only included participants in our repeated-measures analyses who retained scans for both the PLC and the CBDV condition; hence, our final sample included 28 individuals (15 neurotypicals and 13 ASD).

### Statistical analysis

Demographic measures (age, full-scale IQ, time between visits, head motion) were compared using a one-way ANOVA (significance level *p* < 0.05).

First, we tested for differences in baseline (PLC condition) striatal FC between groups using an ANOVA with group as the between-group factor. Given the exploratory nature of this pilot study, we did not correct for multiple comparisons across the 12 seed regions. However, we recognize this is a point of debate and in the interest of transparency, we also include information about whether these effects would have survived highly stringent correction for multiple comparisons across six bilateral (0.05/6 = 0.008) and/or 12 individual (0.05/12 = 0.004) seeds.

Second, we examined whether between-group differences in baseline striatal FC were modulated by pharmacological probing. We used a repeated-measures ANOVA with group as the between-group factor and drug (PLC, CBDV) as the within-group factor, and corrected for multiple comparisons (0.05/3 = 0.017) across the three connections (cluster 1/target 1, cluster 1/target 2, cluster 2/target 1) included in this analysis step. We also conducted supplementary analyses to delineate baseline striatal FC within each group using a one-sample t test. Due to the exploratory nature of our study, these results were not corrected across seeds.

Additionally, to account for multiple comparison corrections, we thresholded our neuroimaging findings at voxel level (*p*_uncor_ < 0.001) and cluster level (*p*_FDR_ < 0.05, cluster-size corr.) [51]. We provide effect sizes (Cohen’s *d*) based on degrees of freedom and T-values of average, group-level, connectivity values within each cluster.

## Results

### Demographics

Our final analysis included 28 individuals (15 neurotypicals and 13 ASD). Groups were matched for age, time between visits, and head motion during both drug conditions (all p ≥ 0.54). In contrast, and as is common in studies including participants with ASD, groups differed slightly in regard to full-scale IQ (*F*(1, 26) = 4.421, *p* = 0.045); specifically, individuals with ASD had slightly lower full-scale IQ (113.0 ± 18.1) than the neurotypical group (125.3 ± 12.7). For more information, please refer to Table 1.

### Striatal FC at baseline and following CBDV

**Fig. 1:**
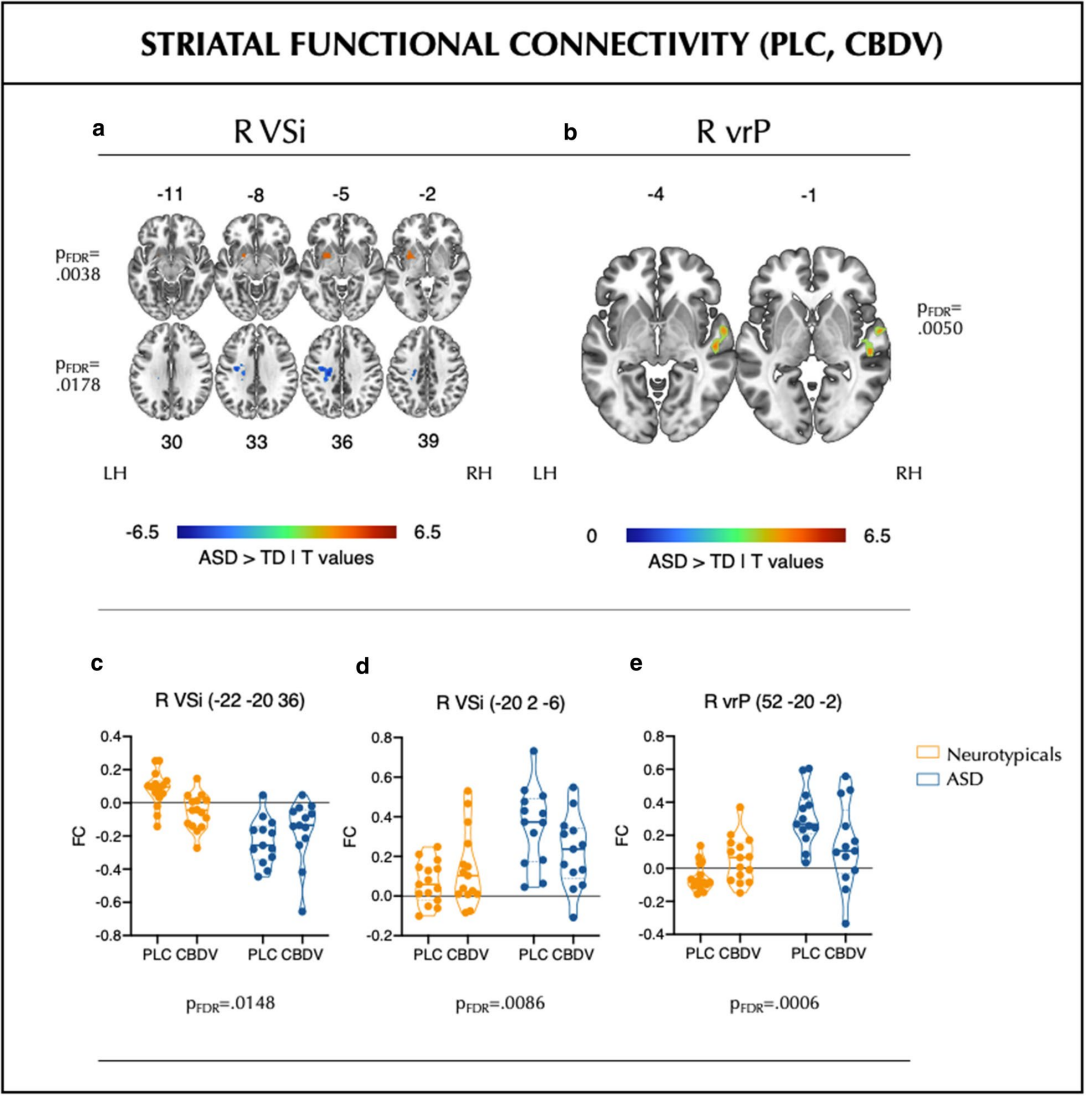
Striatal functional connectivity (FC) after placebo/drug administration. **a**, **b** Baseline between-group (ASD > neurotypicals) differences in FC of the right inferior ventral striatum (R VSi) and right ventral-rostral putamen (R vrP), respectively. Colourbars indicate *T*-values. Numbers above and below slices indicate Montreal Neurological Institute (MNI) *z*-coordinates. **c**–**e**: Group (ASD > neurotypicals) by drug (CBDV > PLC) interaction effects on atypical baseline striatal FC between the R VSi/R vrP and clusters identified above (MNI coordinates represent peak voxel locations). Graphs show average values per subject/cluster. *FC* functional connectivity (Fisher-transformed Pearson’s Correlation coefficients), *L* left, *LH* left hemisphere, *PLC* placebo, *R* right, *RH* right hemisphere, *vrP* ventral-rostral putamen, *VSi* inferior ventral striatum

**Fig. 2:**
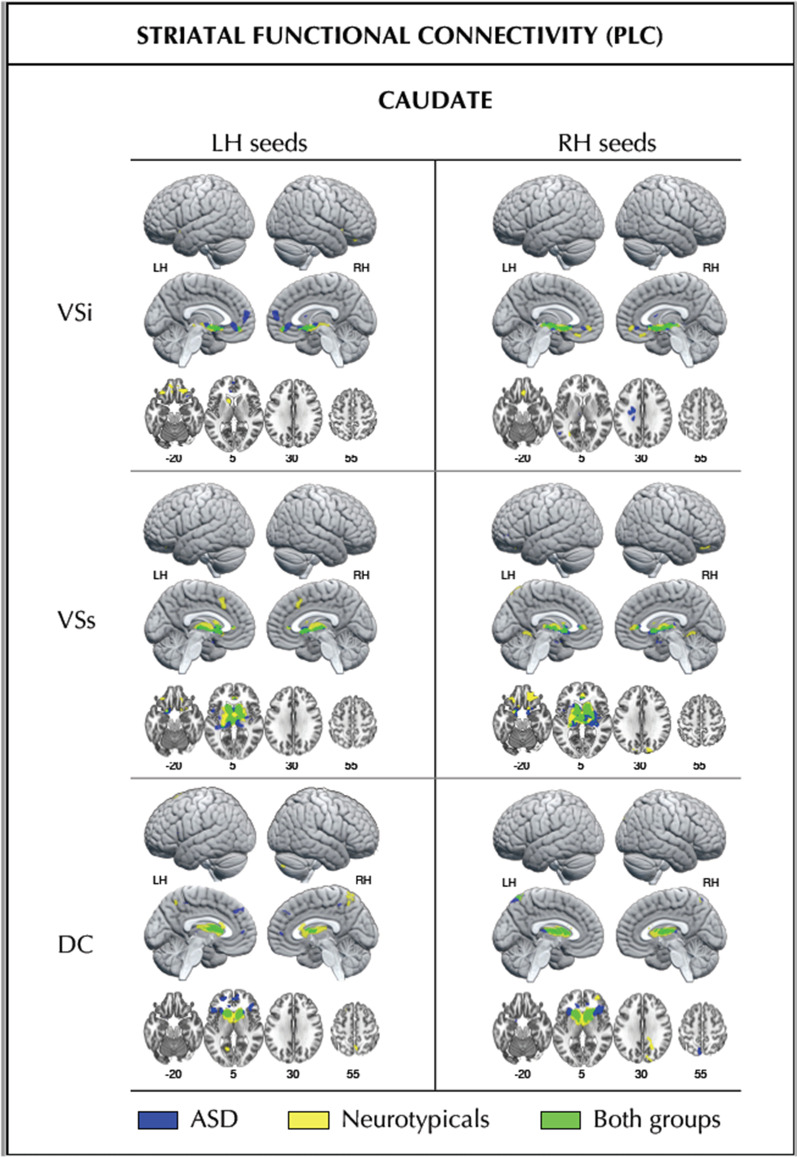
Baseline striatal (caudate) functional connectivity within each participant group. Striatal (caudate) functional connectivity at placebo in the ASD group (blue), the neurotypicals (yellow), and the overlap of both groups (green). Numbers below slices indicate Montreal Neurological Institute (MNI) z-coordinates. *DC* dorsal caudate, *LH* left hemisphere, *RH* right hemisphere, *VSi* inferior ventral striatum, *VSs* superior ventral striatum

**Fig. 3:**
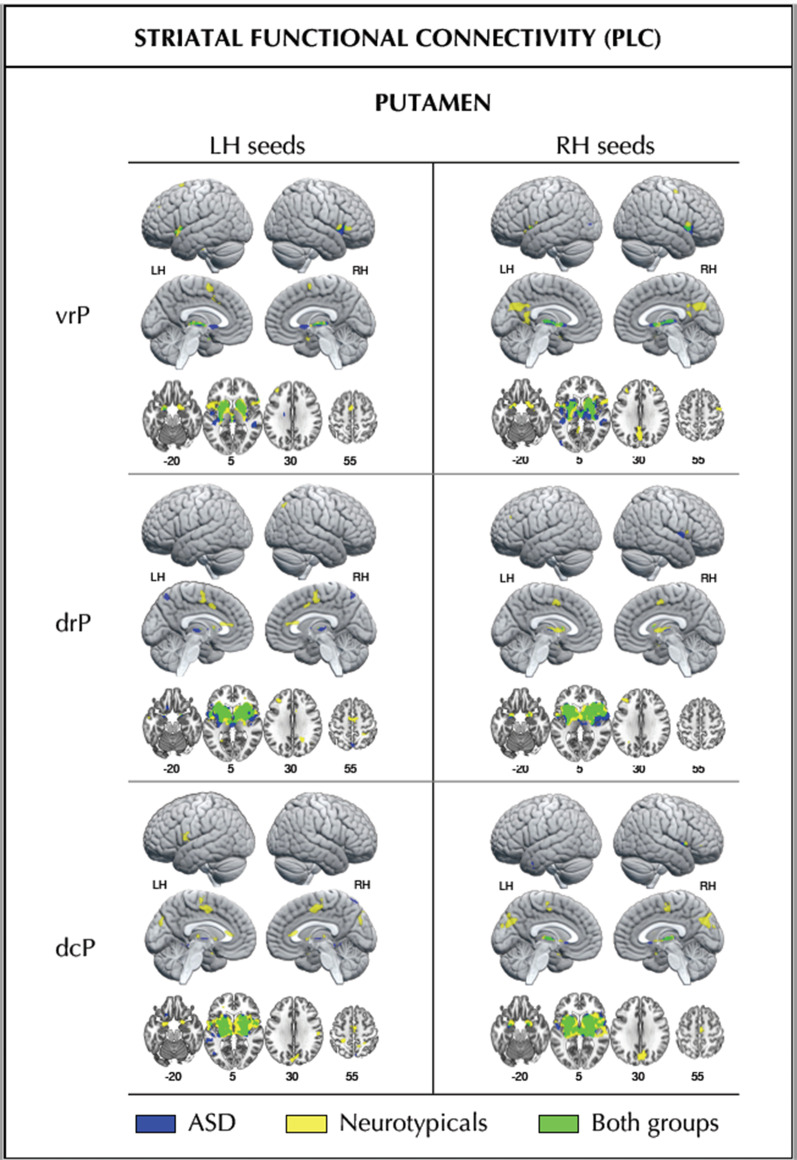
Baseline striatal (putamen) functional connectivity within each participant group. Striatal (putamen) functional connectivity at placebo in the ASD group (blue), the neurotypicals (yellow), and the overlap of both groups (green). Numbers below slices indicate Montreal Neurological Institute (MNI) z-coordinates. *dcP* dorsal-caudal putamen, *drP* dorsal-rostral putamen, *LH* left hemisphere, *RH* right hemisphere, *vrP* ventral-rostral putamen

Next, we identified between-group differences in baseline striatal FC and examined whether these were altered by CBDV. As expected, we observed both striatal hyper- and hypoconnectivity in ASD (Fig. 1, Additional file 1: Table S1). This hyperconnectivity in ASD at baseline was shifted toward neurotypical (no differences between groups during CBDV) following pharmacological probing (Fig. 1, Additional file 1: Table S2). Last, we conducted supplementary analyses to delineate striatal baseline FC within each group separately, the results of which can be found in Figs. 2, 3 and Additional file 2: Tables S3–4. We observed spatially overlapping patterns of FC of groups (ASD vs TD) and hemispheric seeds (left vs right), as well as group- and hemisphere-specific spatial profiles.

### R VSi: left anterior paracentral lobule/left anterior cingulate gyrus

Baseline FC between the R VSi and a cluster containing the left anterior paracentral lobule and left anterior cingulate gyrus was lower in the ASD group compared to the neurotypicals (*p*_FDR_ = 0.0038, Cohen’s *d* = 2.42; this result was retained following multiple comparison correction across 6 bilateral/12 unilateral seeds). We observed a group-by-drug interaction effect on this connection (*p*_FDR_ = 0.0148, *d* = 1.02; this result survived multiple comparison correction across connections). Post hoc testing indicated that, although CBDV somewhat increased FC in the ASD group, this effect was not statistically significant; in contrast, CBDV elicited a statistically significant decrease in FC in the neurotypicals (*p* = 0.0127, *d* = 1.53).

### R VSi: left putamen

Baseline FC between the R VSi and the left putamen was greater in the ASD group compared to the neurotypicals (*p*_FDR_ = 0.0178, *d* = 1.97; result not retained following multiple comparison correction across seeds). Again, we observed a significant group-by-drug interaction effect on this functional connection (*p*_FDR_ = 0.0086, *d* = 1.11; result survived multiple comparison correction across connections). Specifically, CBDV caused a statistically significant reduction in this FC in the ASD group (*p*_FDR_ = 0.0476, *d* = 1.28), but had no effect in the neurotypicals.

### R vrP: right posterior superior temporal gyrus

Baseline FC between the R vrP and the right posterior superior temporal gyrus was greater in the ASD group compared to the neurotypicals (*p*_FDR_ = 0.0050, *d* = 2.50; results retained following correction for six, but not 12, seeds). We observed a group-by-drug interaction effect (*p*_FDR_ = 0.0006, *d* = 1.52; result survived multiple comparison correction across connections). Again, CBDV reduced FC in the ASD group (*p*_FDR_ = 0.0170, *d* = 1.60), but also increased it in the neurotypicals (*p*_FDR_ = 0.0181, *d* = 1.43).

## Discussion

Core and associated symptoms in ASD have been linked to atypicalities in the striatum and its functional circuitry. However, it remains unclear how exactly the striatum is functionally connected—and if its (atypical) FC can be shifted pharmacologically—in ASD. Here we investigated this in a small pilot study using a seed-based approach to resting state functional magnetic resonance imaging (rsfMRI) data. As expected, we found that, in ASD, the striatum displayed both hyper- and hypoconnectivity with numerous cortical regions; and that CBDV shifted atypical connections towards neurotypical findings at baseline.

Previous studies have used detailed seed-based approaches to examine striatal FC, for example, in neurotypical adults [13] and autistic children [32]. Also, prior research has examined the effects of cannabinoids on striatal FC in both neurotypical and patient groups. For instance, CBD has been reported to increase striatal FC with the prefrontal cortex in neurotypicals at rest [52], reduce striatal-hippocampal FC in people with psychosis at rest [53], and increase striatal-frontal FC in occasional cannabis users during a visual oddball task [41]. Here, we extended this research to adults with ASD and to CBDV.

We found that the R VSi and the left putamen were hyperconnected in the ASD compared to the neurotypical group. This finding is similar to previous studies that reported local overconnectivity in ASD [54]. CBDV reduced this atypically strong intra-striatal FC in ASD, while exerting no effects on this connection in the neurotypicals. Similarly, baseline FC between the R vrP and the right posterior superior temporal gyrus was increased in ASD compared to the neurotypicals. Again, CBDV significantly reduced this hyperconnectivity in ASD. The right posterior superior temporal gyrus is thought to support hearing, speech, and language [55, 56]. Together with the vrP, it forms part of a cortico-striatal ‘associative/cognitive’ loop. Hence, our findings are not only in line with previous studies in ASD showing disruptions in striatal-temporal FC [32, 57], they also match reports of impairments in associative and cognitive processing in this condition [58].

**Fig. 4:**
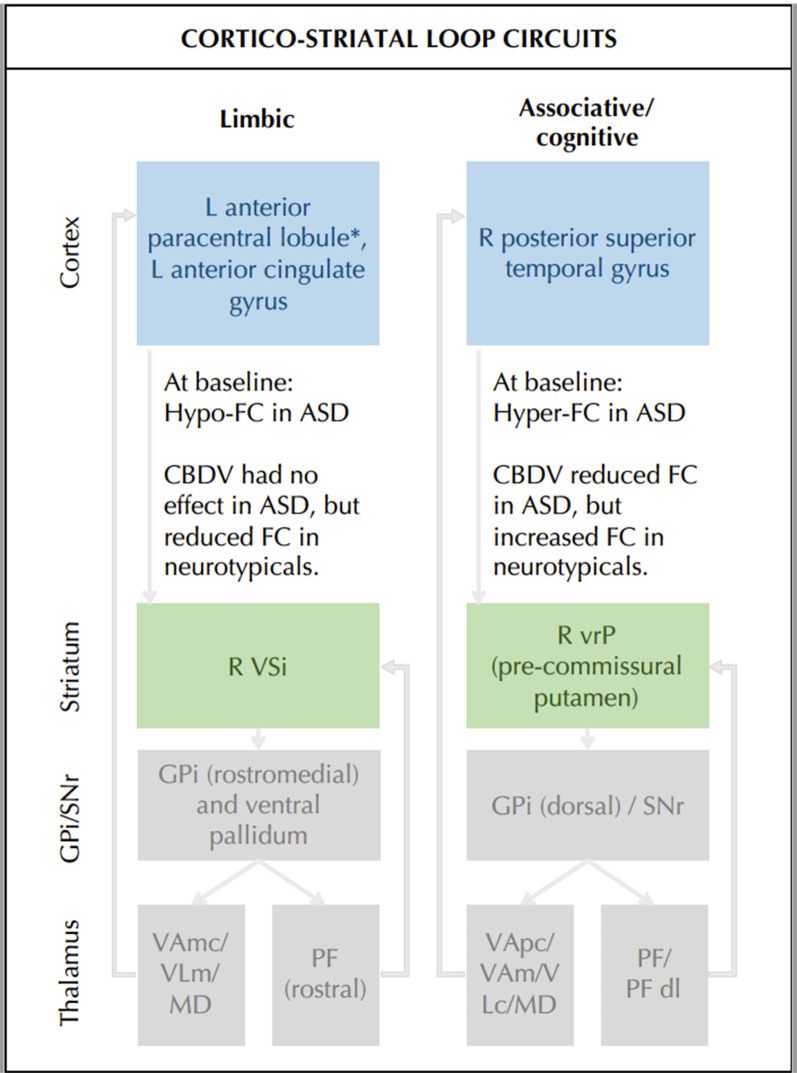
Reported findings in the context of cortico-striatal loop circuits. Baseline differences and group (ASD > neurotypicals) by drug (CBCV > PLC) interaction effects on cortico-striatal loop circuits, including the limbic and associative/cognitive loop, each of which are theorized to include different GPi/SNR and thalamic regions. Grey arrows indicate functional connections. *ASD* autism spectrum disorder, *CBDV* cannabidivarin, *FC* functional connectivity, *GPi* globus pallidus pars interna, *L* left, *MD* mediodorsal nucleus, *PF* parafascicular nucleus of the thalamus (dl: dorsolateral extension), *R* right, *SNr* substantia nigra pars reticulate, *VA* ventral anterior nucleus of the thalamus (mc: magnocellular part; pc: parvocellular part), *VL* ventrolateral nucleus of the thalamus (c: caudal part; m: medial part), *vrP* ventral-rostral putamen, *VSi* inferior ventral striatum. *The anterior paracentral lobule is associated with sensori-motor processing and may represent ectopic FC within the ‘limbic loop’ here

In contrast, we observed weaker FC in ASD relative to the neurotypicals between the R VSi and the left anterior paracentral lobule and left anterior cingulate gyrus. Although not statistically significant, qualitative inspection of the data (Fig. 1c) suggests that in ASD, CBDV tended to increase this FC towards the neurotypical pattern found at baseline. In the neurotypicals, on the other hand, this connection was significantly decreased by CBDV. The anterior paracentral lobule is implicated in vision and motor processing [59, 60]. It is traditionally thought to belong to the ‘sensori-motor loop’, which includes the post-commissural putamen rather than the ventral striatum [61]. Hence, our findings are consistent with previous work in ASD demonstrating atypical striatal FC with (pericentral) regions involved in vision and motor processing [26, 30, 57], and dysfunction in these domains [62–64]. The anterior cingulate is implicated in emotional learning, the expression of emotional states [65], and self-regulation [66]. Together with the VSi, this region is thought to form part of a cortico-striatal ‘limbic loop’ that underpins emotion processing [61, 67]. Our findings of disruptions of this circuitry in ASD match previous reports of atypical fronto-striatal circuitry [26, 27] and emotion processing difficulties [68, 69] in this condition. A schematic of our findings in the context of the mentioned ‘loop’ systems can be found in Fig. 4.

For the sake of completeness, we also conducted supplementary analyses delineating striatal baseline FC within each group separately. Our resulting findings provided a more detailed illustration of striatal FC patterns within each group. They further highlighted similarities and differences in FC profiles between groups, including those that were too underpowered to survive between-group testing). As such, they are to be taken as purely descriptive.

Thus, our findings add to the existing literature by suggesting that (1) in the adult autistic brain, the complex spatial organizational patterns of striatal FC along axes within/between subregions are disrupted. (2) These disruptions affect regions whose functions (e.g. cognitive, affective, associative, language/communication, or motor processing) are implicated in ASD symptoms [58, 62, 70, 71]. Moreover, (3) these striatal systems were responsive to pharmacological probing. A single dose of CBDV was sufficient to shift atypical striatal FC in the mature autistic brain towards the profile found at baseline in neurotypicals.

The precise neurobiological mechanisms underpinning the (differential) effects of CBDV on FC in the two groups are not clear. However, since CBDV has been shown to alter E–I balance in the basal ganglia [18] and the E–I system helps regulate FC [72–75], a shift in striatal E–I may contribute to a shift in striatal FC. In ASD, a wealth of studies suggests that E–I systems are altered [76–80]. This may help explain the differential effects of CBDV on E–I dependent FC. However, the striatum is a hub integrating information from multiple neural regions, many of which may also possess targets for CBDV. Hence, we cannot exclude the possibility that at least some of our results derive from indirect action of CBDV on regions connected with the striatum (e.g. substantia nigra, ventral tegmentum, amygdala, frontal cortex, etc. [81]).

Notably, our study was not designed to determine the impact of CBDV on cognition or behaviour in ASD. However, we suggest that it is possible that the modulation of striatal FC influences the cognitive functions supported by these pathways. Therefore, further examination of whether the biological impact of CBDV on brain is accompanied by, and/or predicts, a clinical response would be a useful next step. For instance, there is evidence from the valproic acid rat model that CBDV impacts on ASD-like behaviours [82]. Combined with the fact that CBDV has a low side effect profile (Investigator’s brochure for CBDV, Edition 4, May 2016), our findings may help develop the rationale for future clinical trials. However, our findings also add to a growing body of literature suggesting that the autistic brain may respond differently to pharmacological probing [18, 83–86], and this should be considered in the development and testing of pharmacological interventions.

## Limitations

Our findings should be viewed in light of several methodological considerations. First, our sample size was relatively small. This was due to difficulties inherent to conducting pharmacological neuroimaging studies in ASD. For instance, these include the necessity to tolerate (mild) discomfort as well as the requirement to stay still during the scan, ingesting the pharmacological compound, navigating study procedures, the absence of medication that may interfere with the tested substance, and readiness to come back for follow-up visits. Nonetheless, we achieved an overall sample size similar to that reported in previous similar studies in ASD [52, 87]. Second, to ensure sufficient power given our limited sample size, we examined a relatively homogeneous group of adult men. However, given that ASD is a neurobiologically heterogeneous condition, our findings should be considered as preliminary and may not ‘generalize’ to others. Future studies should therefore aim to study more heterogeneous samples, e.g. including children, women, and those with co-occurring conditions.

Third, we emphasize some important differences between previous approaches to examine FC in ASD, especially a recent large-scale analysis of whole-brain FC patterns in autistic and neurotypical individuals [88], and our study. Holiga and colleagues’ study was designed to map common baseline FC differences across whole brain in large numbers of people with(out) ASD. Conversely, our study was designed to investigate whether there are differences in the pharmacological *response* to CBDV in striatal FC. Further, Holiga et al. carried out a data-driven analysis of baseline FC using a degree centrality graph metric across the whole brain in children and adults, and males and females. In contrast, our study specifically aimed to examine FC of pre-specified regions of interest using a different FC metric in a small, focused study limited to adult men. This approach precluded us from assessing whether the observed differences in FC (and its responsivity to pharmacological challenge) in ASD were limited to the striatum and its circuitry—or whether they extended to other regions/connections. Therefore, future studies into the effect of CBDV on whole-brain FC should examine larger groups that are suitably powered using data-driven approaches, and also apply alternative statistical tools, such as graph theory. Thus, the FC measures reported previously by Holiga et al. and ours are not directly comparable. Fourth, this study examined the effect of acute CBDV manipulation. This was to establish proof of concept that CBDV can manipulate atypical striatal FC in the autistic and neurotypical adult brain. However, there may be a difference in brain response to a single dose compared to long-term (cannabinoid) pharmacological probing [89, 90]. Accordingly, future studies should examine the effect of sustained CBDV challenge on brain, including striatal FC.

## Conclusion

In conclusion, we identified atypicalities in striatal FC (both hyper- and hypoconnectivity) in adults with ASD. We also provided proof of concept that, in the adult autistic brain, atypical striatal circuitry can be shifted towards a more neurotypical pattern of FC through a single acute dose of CBDV. Future studies should examine whether this modulation of striatal FC in ASD affects cognition and behaviour.

## Supplementary Information

**Additional file 1.** **Figure S1.** Placement of striatal seeds. Numbers above slices indicate Montreal Neurological Institute (MNI) z-coordinates. Abbreviations: A, anterior; DC, dorsal caudate; dcP, dorsal-caudal putamen; drP, dorsal-rostral putamen; I, inferior; L, left; P, posterior; R, right; ROI, region of interest; S, superior; vrP, ventral-rostral putamen; VSi, inferior ventral striatum; VSs, superior ventral striatum. **Table S1.** Between-group (ASD>neurotypicals) differences in striatal functional connectivity at baseline. Table displays statistics for targets (regions containing the cluster peak), including cluster size (in voxels), test statistics, and peak coordinates in Montreal Neurological Institute (MNI) space (x,y,z). Abbreviations: d, Cohen’s d; L, left; R, right; ROI, region of interest; vrP, ventral-rostral putamen; VSi, inferior ventral striatum. **Table S2.** Interaction effect (ASD>neurotypicals, CBDV>PLC) on atypical baseline striatal functional connectivity. Abbreviations: d, Cohen’s d; L, left; R, right; vrP, ventral-rostral putamen; VSi, inferior ventral striatum.

**Additional file 2.** **Table S3.** Baseline striatal functional connectivity in the neurotypicals. Table displays statistics for targets (regions containing the cluster peak), including cluster size (in voxels), T-value (T-val), FDR-corrected significance level (pFDR), and peak coordinates in Montreal Neurological Institute (MNI) space (x,y,z). Abbreviations: DC, dorsal caudate; dcP, dorsal-caudal putamen; drP, dorsal-rostral putamen; L, left; R, right; ROI, region of interest; vrP, ventralrostral putamen; VSi, inferior ventral striatum; VSs, superior ventral striatum. **Table S4.** Baseline striatal functional connectivity in the ASD group. Table displays statistics for targets (regions containing the cluster peak), including cluster size (in voxels), T-value (T-val), FDR-corrected significance level (pFDR), and peak coordinates in Montreal Neurological Institute (MNI) space (x,y,z). Abbreviations: DC, dorsal caudate; dcP, dorsal-caudal putamen; drP, dorsal-rostral putamen; L, left; R, right; ROI, region of interest; vrP, ventralrostral putamen; VSi, inferior ventral striatum; VSs, superior ventral striatum.

## Data Availability

The datasets used and/or analysed during the current study are available from the corresponding author on reasonable request.

## Abbreviations

ASD: Autism spectrum disorder
CBD: Cannabidiol
CBDV: Cannabidivarin
CSF: Cerebrospinal fluid
DC: Dorsal caudate
dcP: Dorsal caudal putamen
drP: Dorsal rostral putamen
E–I: Excitation–inhibition
FC: Functional connectivity
GABA: Gamma amino butyric acid
GM: Grey matter
MNI: Montreal neurological institute
PLC: Placebo
vrP: Ventral rostral putamen
VSi: Inferior ventral striatum
VSs: Superior ventral striatum
WM: White matter
