# Supplementary material for: Modulation of striatal functional connectivity differences in adults with and without autism spectrum disorder in a single-dose randomized trial of cannabidivarin
Source: Mol Autism. 2021 Jul 1;12:49. doi: 10.1186/s13229-021-00454-6 (PMC8252312; doi:10.1186/s13229-021-00454-6)
Supplement: Supplementary file 1 — Additional file 1. Figure S1. Placement of striatal seeds. Numbers above slices indicate Montreal Neurological Institute (MNI) z-coordinates. Abbreviations: A, anterior; DC, dorsal caudate; dcP, dorsal-caudal putamen; drP, dorsal-rostral putamen; I, inferior; L, left; P, posterior; R, right; ROI, region of interest; S, superior; vrP, ventral-rostral putamen; VSi, inferior ventral striatum; VSs, superior ventral striatum. Table S1. Between-group (ASD>neurotypicals) differences in striatal functional connectivity at baseline. Table displays statistics for targets (regions containing the cluster peak), including cluster size (in voxels), test statistics, and peak coordinates in Montreal Neurological Institute (MNI) space (x,y,z). Abbreviations: d, Cohen’s d; L, left; R, right; ROI, region of interest; vrP, ventral-rostral putamen; VSi, inferior ventral striatum. Table S2. Interaction effect (ASD>neurotypicals, CBDV>PLC) on atypical baseline striatal functional connectivity. Abbreviations: d, Cohen’s d; L, left; R, right; vrP, ventral-rostral putamen; VSi, inferior ventral striatum. [file 13229_2021_454_MOESM1_ESM.docx]

**Figure S1 Placement of striatal seeds.** Numbers above slices indicate Montreal Neurological Institute (MNI) z-coordinates. Abbreviations: A, anterior; DC, dorsal caudate; dcP, dorsal-caudal putamen; drP, dorsal-rostral putamen; I, inferior; L, left; P, posterior; R, right; ROI, region of interest; S, superior; vrP, ventral-rostral putamen; VSi, inferior ventral striatum; VSs, superior ventral striatum.

Table S1 Between-group (ASD>neurotypicals) differences in striatal functional connectivity at baseline.

| **ROI** | **Seed** | **Target** | **Size (vox)** | **Statistic** | **x** | **y** | **z** |
| --- | --- | --- | --- | --- | --- | --- | --- |
| Caudate | R VSi | L anterior paracentral lobule,  L anterior cingulate gyrus | 169 | T(26)=-6.17, p_FDR_=.0038, d=2.42 | -22 | -20 | 36 |
|  |  | L putamen | 111 | T(26)=5.01, p_FDR_=.0178, d=1.97 | -20 | 2 | -6 |
| Putamen | R vrP | R posterior superior temporal gyrus | 158 | T(26)=6.38, p_FDR_=.0050, d=2.50 | 52 | -20 | -2 |

Table displays statistics for targets (regions containing the cluster peak), including cluster size (in voxels), test statistics, and peak coordinates in Montreal Neurological Institute (MNI) space (x,y,z). Abbreviations: d, Cohen’s d; L, left; R, right; ROI, region of interest; vrP, ventral-rostral putamen; VSi, inferior ventral striatum.

**Table S2 Interaction effect (ASD>neurotypicals, CBDV>PLC) on atypical baseline striatal functional connectivity.**

| **ROI** | **Seed** | **Target** | **Interaction (ASD>neurotypicals, CBDV>PLC)** | **Within ASD** | **Within neurotypicals** |
| --- | --- | --- | --- | --- | --- |
| Caudate | R VSi | L anterior paracentral lobule,  L anterior cingulate gyrus | T(26)=2.61, p_FDR_=.0148, d=1.02 | ns | T(14)=-2.86, p_FDR_=.0127, d=1.53 |
|  |  | L putamen | T(26)=-2.84, p_FDR_=.0086, d=1.11 | T(12)=-2.21, p_FDR_=.0476, d=1.28 | ns |
| Putamen | R vrP | R posterior superior temporal gyrus | T(26)=-3.88, p_FDR_=.0006, d=1.52 | T(12)=-2.77, p_FDR_=.0170, d=1.60 | T(14)=2.67, p_FDR_=.0181, d=1.43 |

Abbreviations: d, Cohen’s d; L, left; R, right; vrP, ventral-rostral putamen; VSi, inferior ventral striatum.

For Tables S3 and S4, please see the separate excel sheet, as these tables exceed the length of one page.

**Table S3 Baseline striatal functional connectivity in the neurotypicals.** Table displays statistics for targets (regions containing the cluster peak), including cluster size (in voxels), T-value (T-val), FDR-corrected significance level (pFDR), and peak coordinates in Montreal Neurological Institute (MNI) space (x,y,z). Abbreviations: DC, dorsal caudate; dcP, dorsal-caudal putamen; drP, dorsal-rostral putamen; L, left; R, right; ROI, region of interest; vrP, ventral-rostral putamen; VSi, inferior ventral striatum; VSs, superior ventral striatum.

**Table S4 Baseline striatal functional connectivity in the ASD group.** Table displays statistics for targets (regions containing the cluster peak), including cluster size (in voxels), T-value (T-val), FDR-corrected significance level (pFDR), and peak coordinates in Montreal Neurological Institute (MNI) space (x,y,z). Abbreviations: DC, dorsal caudate; dcP, dorsal-caudal putamen; drP, dorsal-rostral putamen; L, left; R, right; ROI, region of interest; vrP, ventral-rostral putamen; VSi, inferior ventral striatum; VSs, superior ventral striatum.
